# Successful treatment of an infected wound involving calciphylaxis via vacuum sealing drainage: A case report

**DOI:** 10.1016/j.ijscr.2023.108008

**Published:** 2023-03-20

**Authors:** Yansheng Jin, Yanrong Zhai, Maoxiao Fan, Xiaozhong Li

**Affiliations:** a Department of Nephrology and Immunology, Children's Hospital of Soochow University, Suzhou, Jiangsu 215025, China; b Department of Nephrology and Immunology, Suzhou Wuzhong People's Hospital, Suzhou, Jiangsu 215128, China; c Department of Orthopedics, Suzhou Wuzhong People's Hospital, Suzhou, Jiangsu 215128, China

**Keywords:** Calciphylaxis, Vacuum sealing drainage, Infected wound

## Abstract

**Introduction and importance:**

The treatment of a complex calciphylaxis wound with infection continues to be a significant challenge in a clinical situation.

**Case presentation:**

We describe a case of an 87-year-old man presented with a nontraumatic painful ulcer on his right heel. Calciphylaxis was diagnosed by the biopsy. Although a serial therapy of sodium thiosulphate, tramadol hydrochloride, frequent dressing changes, and conventional surgical debridement initiated, the worsening skin wound covered with a necrotic base and blackish eschar was formed. Skin cultures returned positive for the growth of *proteus mirabilis*. Subsequently, the usage of vacuum sealing drainage appeared to be effective. A complete resolution occurred 12 weeks after vacuum sealing drainage therapy.

**Clinical discussion:**

The infected wound due to calciphylaxis is not uncommon. Vacuum sealing drainage technique demonstrated the effective approach to deal with wound infections, especially in the early stage. As the initial measures of wound care, serial debridement and sodium thiosulphate treatment have not slowed down the progress of the disease in this case, the vacuum sealing drainage along with the creative use of topical antibiotic flushing provided the best solution to promote healing of the infected field.

**Conclusion:**

This case highlights that vacuum sealing drainage technique could be considered in patients presenting with an infected wound involving calciphylaxis.

## Highlights

• Treatment of an infected wound involving calciphylaxis is a challenging in clinic.
• Vacuum sealing drainage promoted the healing of infected calciphylaxis wound.
• Vacuum sealing drainage is considered in infected wound involving calciphylaxis.

## 1 Introduction

Calciphylaxis, commonly known as calcific uremic arteriolopathy, is a fatal disorder characterized by ischemic tissue necrosis due to small vascular calcifications [1]. Patients clinically present with painful skin lesions that evolve into nonhealing wounds in a clinical situation [2]. Resultant wounds can cause a systemic inflammatory response, which resulting in a severe infected wound and sepsis that is considered the most common cause of death [3].

As limited evidenced of treatment options and guidelines, the management of a complex calciphylaxis wound with infections is exceeding difficult. To our knowledge, active wound treatment with vacuum sealing drainage (VSD) therapy is considered a vital important modality to accelerate wound healing and improve prognosis. VSD maybe a worthy of clinical application for infected wound associated with calciphylaxis, although warrant further researches.

This case report has been produced in keeping with the SCARE criteria [4].

## 2 Presentation of case

**Fig. 1:**
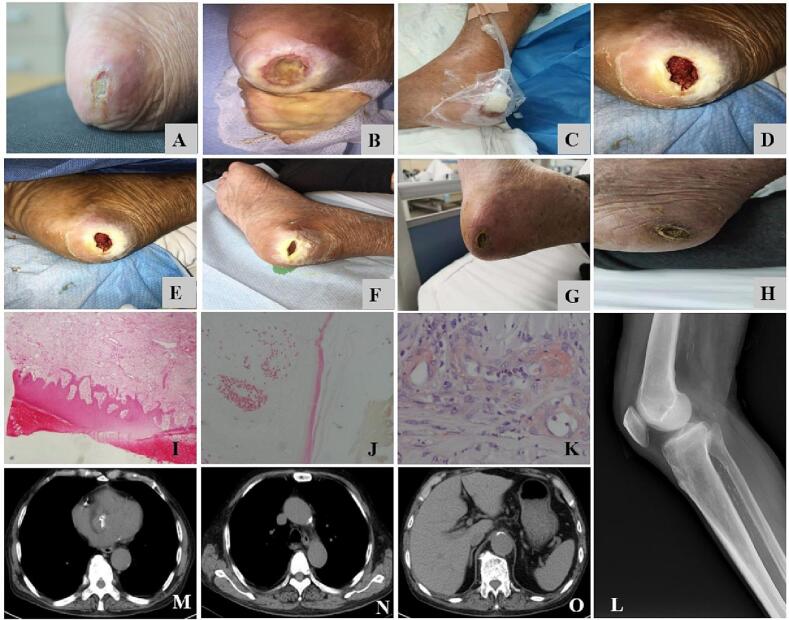
Clinical manifestations, radiographic features, and histologic characteristics of calciphylaxis. Panels A and B show the early worsening manifestations of calciphylaxis: a swelling, pale and tender ulcer (Panel A), a malodorous ulcer with a necrotic base and overlying black eschars (Panel B). The vacuum sealing drainage (VSD) was conducted to reduce infection wound involving Calciphylaxis (Panel C). Panels D through H show the Clinical Images after the Use of VSD: 5 days after treatment (Panel D), 30 days after treatment (Panel E), 40 days after treatment (Panel F), 70 days after treatment (Panel G), 3 Months after treatment (Panel H). A skin-biopsy section stained with hematoxylin and eosin shows a small amount of lymphomonocyte infiltration in the superficial dermis (10 × 10, Panel I), von Kossa stain reveals a negative calcification of arteriolar (Panel J), alizarin red staining displays fine arteriolar calcifications (40 × 10, Panel K). Plain radiograph of right lower extremity shows multiple patchy calcifications in soft tissue (Panel L). Computed tomography scan shows multiple calcifications occurring in coronary artery (Panel M), aortic valve (Panel M), ascending aorta (Panel N), and aortaventralis (Panel O).

An 87-year-old man was admitted with the chief complaint of a painful non-trauma nonhealing right heel skin lesion for 2 weeks duration. His past medical history included hemodialysis for 6 years with chronic glomerulonephritis as the presumed cause of end-stage renal disease. On transfer to our department, the patient's wound was comprehensively assessed. In the physical examination, an obvious painful right heel ulceration, approximately 1.5 cm in length, that was embedded in the surrounding tissues, with underlying tissue necrosis, were observed. The peripheral sensation of the right lower limb was normal, the peripheral blood supply of right lower limb was good, and right dorsalis pedis artery and posterior tibial artery pulsation was touched. A dermatology consultation noted areas of debrided tissue coupled with yellow rotting flesh with purulent secretion accompanied by putrefactive odor and conventional surgical debridement was recommended. The experienced dermatologist conducted initial debridement of the necrotic tissue and some deeper subcutaneous tissues taken from the lesion margin were obtained for biopsy. Pathology of the lesion showed a small amount of lymphomonocyte infiltration in the superficial dermis and fine arteriolar calcifications most consistent with calciphylaxis (see Fig. 1). Multiple calcifications occur in coronary artery, aortic valve, ascending aorta and aortaventralis by computer tomography in this case (see Fig. 1). Radiographic imaging is nonspecific but can demonstrate soft tissue and vascular calcifications in advanced disease states. Pertinent laboratory works were presented (see Table 1).

**Table 1:** Pertinent laboratory works.

Values	On admission	Before VSD treatment	After VSD treatment	Reference range
White blood cell (×10^9^/L)	5.92	8.33	5.61	4–10
Neutrophilic granulocyte percentage (%)	63.50	85.70	67.30	50–70
Blood platelet (x10^9^/L)	256.00	259.00	261.00	100–300
Hemoglobulin (g/L)	130.00	126.00	125.00	120–175
Albumin (g/L)	32.70	31.00	32.40	37–53
Serum calcium (mmol/L)	2.18	2.24	2.14	2.08–2.8
Serum phosphatase (mmol/L)	1.45	1.49	1.54	0.8–1.6
Blood urea nitrogen (mmol/L)	21.43	28.43	24.54	2.9–8.2
Serum creatinine (μmol/L)	889.2	893.4	897.5	59–104
β2-microglobulin (mg/L)	15.00	18.00	17.40	0–2.7
Parathyroid hormone (pg/mL)	267.1	N	268.7	15–65
Procalcitonin	0.12	0.14	0.08	<0.1
Erythrocyte sedimentation rate (mm/1 h)	25	43	26	0–15
Hypersensitive C-reactive protein (mg/L)	0.89	61.44	1.58	0–3
Interleukin −10 (pg/mL)	<5	13	<5	≤9.1
Tumor necrosis factor-α (pg/mL)	27.30	47.40	28.34	≤8.1
Interleukin −6 (pg/mL)	7.18	18.40	8.45	≤5.9
Serum 25-hydroxyvitamin D (ng/mL)	25.81	N	26.48	20–100
Culture of wound secretion	Negative	*Proteus mirabilis* (4+)	Negative	Negative

According to the clinical manifestations, radiographic features, and histologic characteristics, calciphylaxis was confirmed (see Fig. 1). A thorough diagnostic workup revealed no signs of infection. The initial treatment included administration of intravenous sodium thiosulfate (STS, 6.4 g once a day) and orally tramadol hydrochloride (0.1 g once a day) with frequent dressing changes and surgical debridement. On account of the lesions were extremely painful, making the debridement difficult. When duration of STS usage for 20 days, the patient appeared with unbearable nausea and vomiting owing to the side-effect of STS, which was discontinued accordingly. Concurrently, an extensive painful ulceration covered with a necrotic base and blackish eschar was formed (see Fig. 1).

The laboratory tests revealed an elevated inflammatory parameters of erythrocyte sedimentation rate, hypersensitive C-reactive protein, interleukin-6, interleukin-10, and tumor necrosis factor-α (see Table 1). Cultures of wound secretion returned positive for the growth of *proteus mirabilis*. The novel technique of vacuum sealing drainage (VSD), relating to an anti-blockage flushing drainage tube along with pipelines using target antibiotic of gentamicin to manage the infected wound involving calciphylaxis, was applied. Over the subsequent 12 weeks, the painful wound was gradually resolved with no recurrence of his disease activity at his 26-month follow up (see Fig. 1).

## 3 Discussion

The infected wound due to calciphylaxis is not uncommon. VSD technique demonstrated the effective approach to deal with wound infections, especially in the early stage [5]. VSD strategy carried a risk of failure in the infections sustained by high-virulence bacteria; however, the calciphylaxis infected wound of the intractable condition in our patient healed successfully. In our view, the complete assessment of patient's overall clinical characteristics is of great importance. Firstly, the infected wound of calciphylaxis occurring after the skin biopsy, wound culture demonstrating *proteus mirabilis*, and the wound exposure were bad prognostic markers in our patient. Secondly, the initiation treatment of surgical debridement, wound care, and STS had failed to handle the infected wound involves calciphylaxis. Ultimately, as the poor situation of the wound infected by the growth of *proteus mirabilis*, the treatment including novel VSD along with topical antibiotic flushing was conducted simultaneously.

The management of a calciphylaxis wound with infection continues to be a vital challenge for clinicians. Several studies have shown that VSD technique has a promising performance in treating wound beds infected with various pathogenic microorganisms [5], [6]. The adjuvant treatment of VSD may help to cover the dead space, reduce wound healing time and the risk of recurrent infection [7]. As the initial measures of wound care, serial debridement and STS treatment have not slowed down the progress of the disease in this case, the VSD along with the creative use of topical antibiotic flushing provided the best solution to promote healing of the infected field within 12 weeks in this case. Recently, Roberson et al. presented a case of extensive calciphylaxis wounds of the bilateral lower extremities complicated by angioinvasive coinfection in a young female patient and a complete resolution of the lesion with almost tissue coverage occurred through a multidisciplinary approach within 26 weeks [8]. Our case highlights that VSD therapy is effective for infected wounds and healing was faster if the wound infection was completely removed. While the underlying mechanisms and biological effects of the VSD on the infected wound involving calciphylaxis warrant further researches, it is possible conduct that VSD maybe a worthy of clinical application to improve wound healing and avoid the serious infection.

Supportive management methods like wound care and analgesia have been described above in our case. Importantly, on the basis of expert opinions, a multidisciplinary approach is often recommended to manage with calciphylaxis [9]. Various treatment options such as risk factors mitigation, optimize dialysis clearance, parathyroidectomy, hyperbaric oxygen therapy, nutrition management, fluids and electrolyte balance may also be essential in these patients for further improvement [10]. Administer systemic medical therapies including sodium thiosulfate, bisphosphonates, tissue plasminogen activator, vitamin K, and bosentan are certainly promising approaches [3]. Nonetheless, the evidence in support of these approaches is predominantly based on observational studies; for this reason, more and more researches on treatment are now underway.

## 4 Conclusion

The management of a complex calciphylaxis wound with infection continues to be a significant challenge in a clinical situation. To our knowledge, active wound treatment with VSD therapy is considered a vital important modality to accelerate wound healing and improve prognosis. Herein, we describe a nonhealing infected wound involving calciphylaxis in a hemodialysis patient, who was successfully resolved by VSD therapy. VSD maybe a worthy of clinical application for infected wound associated with calciphylaxis, although warrant further researches.

## Sources of funding

This work was supported by Science and Technology Development Plan of Suzhou (SS202067, SKYD2022072).

## Consent

In this work, the written informed consent was provided by the patient to report the case images and other details clinical information. A copy of the written consent is available for review by the Editor-in-Chief of this journal on request.

## Ethical approval

This study has been approved by the ethics committee of Suzhou Wuzhong People's Hospital (2022xjs010).

## Author contribution

Dr. Yansheng Jin and Dr. Maoxiao Fan were involved in the writing of this case report. Yansheng Jin and Yanrong Zhai joined the vacuum sealing drainage therapy. P. Xiaozhong Li revised the paper. Yansheng Jin, Yanrong Zhai and Maoxiao Fan contributed equally to this work.

## Registration of research studies

This case report is not a “First in Man” and therefore does not require any registration.

## Guarantor

Dr. Yansheng Jin M.D. and Dr. Xiaozhong Li, M.D.

## Declaration of competing interest

There are no conflicts of interest in the creation of this case report as declared by the authors.
